# Participative Leadership: A Literature Review and Prospects for Future Research

**DOI:** 10.3389/fpsyg.2022.924357

**Published:** 2022-06-03

**Authors:** Qiang Wang, Hong Hou, Zhibin Li

**Affiliations:** ^1^School of Economics and Management, University of Science and Technology Beijing, Beijing, China; ^2^College of Business Administration, Gachon University, Seongnam, South Korea; ^3^School of Management, Xi’an Polytechnic University, Xi’an, China

**Keywords:** participative leadership, employee, participation, leadership, organization, decision, effectiveness

## Abstract

Changes in the external market environment put forward objective requirements for the formulation of organizational strategic plans, making it difficult for the organization’s leaders to make the right and effective decisions quickly on their own. As a result, participative leadership, which encourages and supports employees to participate in the decision-making process of organizations, has received increasing attention in both theory and practice. We searched the literature related to participative leadership in databases such as Web of Science, EBSCO, ProQuest, and China National Knowledge Infrastructure (CNKI). Based on this, we clarify the concept of participative leadership, propose a definition of participative leadership, summarize measurement scales for this type of leadership, and compare participative leadership with other leadership styles (empowering leadership and directive leadership). We also present a research framework for participative leadership that demonstrates its antecedents; the mechanisms for its development based on social exchange theory, conservation of resources theory, social cognitive theory; social information processing theory, and implicit leadership theory; and outcomes. Finally, we identify five potential research areas: Connotation, antecedents, outcomes, mediators and moderators, and study of participative leadership in China.

## Introduction

In the digital age, companies are actively taking accurate decisions such as using advanced technology to enhance their competitive advantage in the marketplace (Su et al., 2021). But where do good measures and perfect solutions come from? The answer comes from the masses. With the dramatic changes in the competitive business environment, it is difficult for organizational leaders to make timely and effective decisions on their own, which has led to the active presence of employees in organizational decision-making today (Peng et al., 2021). At the same time, due to the use of modern information technology such as computer networks and system integration, there is a bottom-up flow of information within the enterprise, and these cross-level, multi-dimensional “employee opinions” play an increasingly important role in leadership decision-making. Improving a company’s competitive advantage, sustainable development goal and performance is increasingly dependent on the active participation of the organization’s employees in decision-making (Chang et al., 2021; Jia et al., 2021). In particular, Peter Drucker, the master of manageme, also considered that “encouraging employee involvement” is an important part of effective leadership in his influential study “Management by Objective.” In practice, some well-known companies have gradually started to call for employee participation behaviors in decision-making to varying degrees. For example, leaders in the R&D department of Volvo Cars actively use shared open rights and encourage diversity initiatives to promote employee participation in decision-making to facilitate organizational innovation (Jing et al., 2017). It is easy to see that employee participation, a key component of organizational decision-making, is an important influencing factor for business organizations to adapt to the dynamic business environment and improve the effectiveness and science of leaders’ decisions. Therefore, it is an important issue that leaders need to focus on in real-time, especially in organizations with a high power distance culture, to promote the participation of their subordinates in organizational decision-making (Huang et al., 2010). This requires leaders to adopt a supportive, democratic leadership style, known as participative leadership. A large number of scholars also agree that organizational leaders are increasingly relying on highly engaged employees to meet the challenges of a competitive marketplace, so participative leadership, which seeks to promote behaviors that support employee participation in organizational decision-making, is gaining attention in many organizations (Huang et al., 2006). Participative leadership exists in organizations of any size, of any type and at any stage, where openness and empowerment of employees in the organizational decision-making process are core characteristics that distinguish it from other leadership styles (Huang et al., 2021). When making strategic decisions, participative leaders are able to share decision-making power and fully consult employees to jointly deal with the work problems (Chan, 2019).

In summary, participative leaders encourage and support employees to participate in the decision-making process in order to make effective organizational decisions and to solve work problems together through a range of measures (Kahai et al., 1997). However, there is still much space for theoretical research on participative leadership, and the organizational practice with the current call for “employee participation in decision-making” needs to be optimised and improved, and there is an urgent need to balance the organizational practice and theoretical research on “employee participation” and “scientific decision-making” from the leadership level. In order to accelerate the exploration of participative leadership and promote the research on the effectiveness of participative leadership, we systematically review the literature on participative leadership, summarise and outline its concept, measurement scales and conceptual comparisons, antecedents, mechanisms and outcomes, and present future research perspectives.

## Method

### Literature Collection

We searched the literature on participative leadership published in databases such as Web of Science, ProQuest, EBSCO, and China National Knowledge Infrastructure (CNKI). To perform the search, we used the keywords “participative leadership,” “participative management,” “participative behavior,” and “participative leader.” We also used a snowballing approach to identify relevant literature by searching the list of references we found in our research. Also, to better examine the similarities and differences in leadership styles in our work, we had collected literature related to directive leadership and empowering leadership in these databases. And we only used the keywords “directive leadership,” and “empowering leadership.”

### Literature Processing

Literature was included in our research if it met the following criteria. First, we collected research on the topic of participative leadership, excluding leadership research unrelated to participative management. Second, the literature we collected on participative leadership had to be written in either English or Chinese, excluding relevant research in other languages. Third, the literature included both quantitative and qualitative research and did not impose any restrictions on where the research was conducted or the industry in which it was conducted. Fourth, the information we collected on participative leadership included published journal articles, conference papers, master’s and doctoral dissertations, and so on. In addition, compared to participative leadership, we collected mostly review-based literature on empowering leadership and directive leadership, including some empirical researches, to better understand both types of leadership. Also, the literature must be written in Chinese or English.

## The Concept of Participative Leadership

According to literature review, participative leadership is a democratic leadership that involves subordinates in organizational decision-making and management, with the aim of effectively enhancing employees’ sense of ownership and actively integrating their personal goals into organizational goals. Therefore, in the daily leadership process, leaders actively implement “participation management” for their subordinates, such as conveying meaningful values, actively organizing reporting and other flexible promotion strategies (Jing et al., 2017). The American scholar Likert (1961), after extensive experimental research on democratic leadership, formally introduced the concept of participative leadership in his book “A New Model of Management” and revealed the three main principles of participative leadership theory, including the mutual support principle, the group decision principle and the high standards principle. Since the introduction of participative leadership, it has received much attention from a large number of researchers. Based on previous research, Kahai et al. (1997) redefined it as participative leadership, which refers to a leadership style in which leaders ask employees for their opinions before making decisions, delegate decision-making authority to subordinates in practice, and encourage active participation by employees to make decisions together. The literature also reflects two core characteristics of participative leadership: first, employees are consulted before decisions are made in order to solve problems together; second, employees are given resources to support them in the work process (Kahai et al., 1997; Lam et al., 2015; Li et al., 2018).

Participative leadership is also characterised in practice by the following features: first, in the process of employee participation in decision-making, leaders and subordinates are on an equal footing and trust each other completely, and organizational issues are resolved through democratic consultation. Second, in general, although participative management involves a wide range of employees in decision-making, the final decision is still made by the leaders. Huang et al. (2010) also explored participative leadership in-depth and argued that participative leadership requires more encouragement and support for employees in the decision-making process and sharing of information and ideas, which has been recognized by many scholars (Xiang and Long, 2013; Lam et al., 2015; Li et al., 2018). It is easy to see that the core of participative leadership is to encourage employees to participate in organizational decision-making, and the key to the leadership process is to make a series of management tasks such as consulting employees before making decisions (Benoliel and Somech, 2014). Thus, based on many previous studies and practical experience, we consider participative leadership as a set of leadership behaviors that promote subordinates to participate in decision-making by giving them a certain degree of discretionary powers, effective information and other resources, as well as care and encouragement, so that they can be consulted enough before making decisions to solve work problems together(Huang et al., 2010; Chan, 2019).

## Measurement of Participative Leadership

The current measurement of participative leadership is mainly in the form of questionnaires in quantitative research and consists of the following measurement scales. First, Vroom (1959) psychological participation questionnaire, which evaluates the frequency with which leaders demonstrate a participative leadership style and reflects the overall ability of members to influence decisions and provide input and advice to leaders, consists of four questions (α = 0.63), sample item: “If you had a suggestion to improve your work or change a process in some way, how easy would it be for you to communicate the idea to your leader.”

Second, the empowering leadership scale (ELS) developed by Arnold et al. (2000) in which subjects score perceived leadership behaviors, with several items in the participation in decision-making section becoming a measure of participative leadership (α = 0.86), and is currently recognized by most scholars, with a total of six questions, and a sample item is “Encourages work group members to express ideas/suggestions.” The measurement scale developed by Arnold et al. (2000) has been widely used in empirical research (Huang et al., 2010; Lam et al., 2015; Peng et al., 2021).

Third, the participative management questionnaires. In research of participative management in education, Somech (2002) designed a participative management scale with a total of thirty-five items, which includes five dimensions: decision domain (10 items; α = 0.83), degree of participation (4 items; α = 0.79), structure (3 items; α = 0.79), rationale (9 items; α = 0.77), and participation target (9 items; α = 0.69). Decision domain refers to determine if, after a decade of explicit attention to and advocacy of enhanced participative leadership, principals prefer to involve teachers not only in the technical domain, but also in the managerial, and a sample item is “Setting and revising the school goals.” Degree of participation refers to differentiating the extent of participation from the degree of participation, and a sample item is “Makes decisions on his or her own.” Structure refers to the extent to which a formal structure for validating decisions exist in the school and their relationship to other dimensions of participation, and a sample item is “To what extent explicit procedures existed at the school concerning who participated in the decision-making process.” Rationale is to determine, through an exploratory method, the main motives that inspired principals to participate in management and their relationship with the degree of participative management, and a sample item is “Encourage teacher’s acceptance of the decision.” Participation target refers to examine principals’ considerations in choosing which teacher to involve in the decision-making process, and a sample item is “The teacher expressed an independent thinking style.” The measurement scale developed by Somech (2002) has been found to have a good use in research (Benoliel and Somech, 2014).

Fourth, some scholars had adapted or developed participative leadership scales by themselves, but the use is limited. For example, Kahai et al. (2004) used group-level responses (3 items) about how frequently participants observed the leader to implement participative management. A sample item is “Incorporating their suggestions into the final decision.” And Li et al. (2018) adapted from Oldham and Cummings (1996) and Kahai’s studies (Kahai et al., 2004), which asked employees to rate their team leaders’ participative leadership behaviors on a four-item scale (α = 0.81), with typical questions such as “Puts suggestions from our group members into the final decision.” The individual responses were aggregated to the team level. Mean*r*_*wg*_ was 0.90. And Zhao et al. (2019) developed a five-item scale, with typical questions such as “Leaders encourage team members to be active in suggesting ideas” (α = 0.80). In addition, there are also some studies that utilize the case method in qualitative research. For example, Jing et al. (2017) used an embedded case approach to provide an in-depth analysis of the role played by participative leadership. Finally, we summarize the major ways and references of previous measurements in the form of tables, as shown in Table 1.

**TABLE 1:** Summary of measurements.

Measurement methods	Major ways	References
Psychological participation questionnaire	Assess how often leaders demonstrate an engaged leadership style	Vroom (1959)
Empowering leadership scale	Subjects score perceived leadership behaviors	Arnold et al. (2000)
Participative management questionnaires	Decision domain, degree of participation, structure, rationale, and participation target	Somech (2002)
Others	Participants observe the frequency with which leaders implement participatory management or rate their team leaders’ participative leadership behaviors	Kahai et al. (2004), Li et al. (2018)

## Comparison Between Participative Leadership and Other Leadership Styles

A review of the recent literature reveals that some scholars usually discuss participative leadership together with empowering leadership and directive leadership, but they are only mentioned, without in-depth analysis of the similarities and differences between them (Lonati, 2020; Zou et al., 2020). At present, the lack of comprehensive comparative analysis of the three leadership styles. Therefore, we analyze the similarities and differences between participative leadership and empowering leadership and directive leadership to varying degrees and compares them in terms of key characteristics, behavioral approaches and behavioral motives to highlight the unique research value of participative leadership, as shown in Table 2.

**TABLE 2:** Contrast of different leadership styles.

Leadership styles	Key characteristics	Behavioral approaches	Behavioral motives
Empowering leadership	Leaders’ behavior toward power-sharing, delegation and employees’ perceptions of empowerment	Management practice measures for delegating authority (personal authority and job responsibilities) to subordinates	Eliminate employees’ inherent sense of disempowerment, achieve employee motives and improve employee performance
Directive leadership	Organize the work of subordinates by giving clear instructions and expectations	Clarify policies, rules, procedures and methods for assigning work tasks and complete them in the form of one-way orders to subordinates	Create a sense of discipline and responsibility and enable employees to focus on specific work tasks
Participative leadership	Encourage employee participation in organizational decision-making	Provide employees with a degree of discretion, effective information and support from other resources, and provide care and encouragement to facilitate their participation	To promote a sense of ownership, so that employees see themselves as responsible for achieving organizational goals, making effective organizational decisions and working together to solve work problems

### Empowering Leadership

The situational empowerment perspective emphasizes the practice of empowerment in organizational situations and defines empowering leadership as a series of management practices that empower subordinates. The psychological empowerment perspective emphasizes the psychological experience of empowerment and defines it as a motivational tool to eliminate employees’ internal feelings of disempowerment by raising their level of motive. And the integration of situational perspective and psychological perspective emphasizes the leaders’ behavior toward power sharing and employees’ perceptions of empowerment, illustrating the process of achieving power sharing between leaders and employees (Tang et al., 2012). It is easy to see that both empowering leadership and participative leadership denote the delegation of leadership authority, but the focus are different. Specifically, participative leadership refers to the sharing and delegation of decision-making power, which means that subordinates are able to participate in the leaders’ decisions and express their views, while empowered leadership is more concerned with the delegation of personal authority and job responsibilities, so that subordinates have a certain degree of autonomy in deciding how to work, in order to achieve self-motive (Amundsen and Martinsen, 2014). In addition, empowering leaders have a certain degree of positivity when they delegate their power, but they also tend to make employees feel that the leader is not willing to manage, which reduces the effectiveness of leadership. However, the participative leaders only share decision-making power with subordinates, retaining the authority and responsibility for leadership work and effectively avoiding employees’ perceptions of laissez-faire management. Thus, participative leadership is unique in that it not only achieves performance goals but also reduces the corresponding negative impacts (Zou et al., 2020).

### Directive Leadership

Directive leadership is about providing specific instructions to employees and clarifying policies, rules and procedures designed to organize the work of subordinates by providing obvious instructions and expectations regarding compliance with instructions (Li et al., 2018; Lonati, 2020). In short, directive leadership is the use of leadership authority to tell subordinates what to do by way of orders, instructions, etc., in order to successfully achieve organizational goals. In other words, directive leadership is the procedure and method by which the leader assigns organizational tasks to subordinates and accomplishes them by means of one-way communication, and there is a relationship of command and obedience, instruction and execution between the leader and subordinates. Not only that, organizations with directive leadership are more likely to have normalized work processes, and employees are likely to obey the precise orders of the leader, allowing themselves to be fully focused on completing specific work tasks (Lorinkova et al., 2013). Consequently, social messages such as clear work objectives, specific work procedures and supervision by organizational leaders create a sense of rules and responsibility among subordinates, but undermine employee creativity. Participative leaders, however, actively engage in interpersonal interaction with their employees in order to make decisions together. And, participative leadership, characterised by autonomy, collaboration and openness, encourages the employees to work innovatively by providing creative ideas and solutions that lead to the best decisions (Lam et al., 2015). Therefore, participative leadership is more effective in stimulating employee creativity than directive leadership.

## Research Framework for Participative Leadership

**FIGURE 1:**
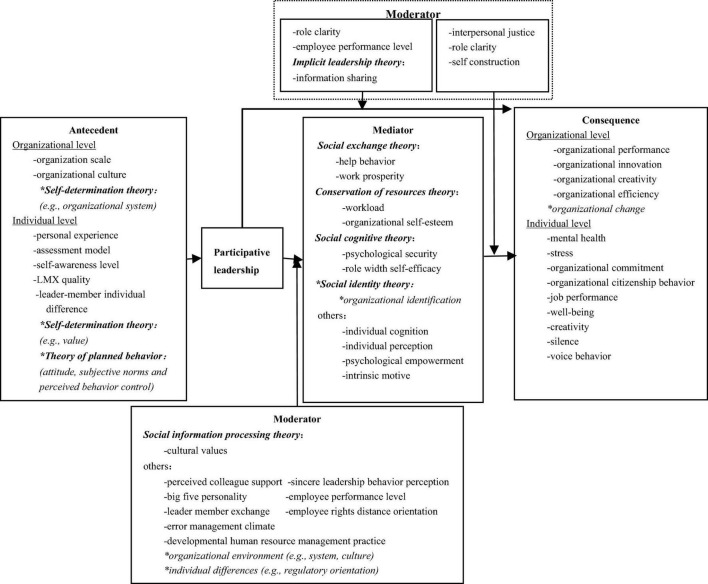
Empirical research on participative leadership. Data sources were reviewed according to relevant literature; “-”represents the existing research path and variables; “*”represents the path and variables proposed in future research.

Changes in the external marketplace put forward objective demands on the development of the organization’s strategic solutions, making it difficult for the organization’s leaders to make the right and effective decisions quickly on their own (Li et al., 2018; Zhao et al., 2019). Based on a review of previous research, we develop a research framework for participative leadership (shown in Figure 1) including the antecedents, mechanisms (mediator and moderator), and consequences of this type of leadership, with a view to clearly showing the lineage of empirical research on participative leadership for scholars’ subsequent exploration.

### The Antecedents of Participative Leadership

The antecedents of participative leadership can provide positive guidance for the development of this leadership research. Currently, the antecedents of participative leadership can be divided into individual-level antecedents and organizational-level antecedents. A lot of studies on antecedents focus on the individual level, such as individual experience, assessment model and leader-member individual difference (Somech, 2002; Li et al., 2018). These factors promote leaders to show more participative management behaviors. In contrast, greater organizational control over participative behaviors tends to push leaders to highlight the significance of employee participation in organizational decision-making. As proof, organizational culture and organizational size have great influence on leaders’ participative management behaviors.

#### Individual-Level Antecedents

Some scholars pointed out that the implementation of participative management is related to personal factors. For example, experienced leaders may be more inclined to engage in participative management (Somech, 2002). Among the specific research on individual influences, the influence of personality tendencies on leadership style has become a key theoretical concern. In particular, based on the regulatory model theory, Li et al. (2018) found that the assessment model refers to the fact that individuals are more concerned with obtaining the best solution during self-regulation, and it is more likely to develop a participative leadership style, while the locomotion model is more concerned with state change and more likely to develop a directive leadership style. At the same time, the leader’s awareness of participative management is key to influencing his or her participative management style and is seen as a determinant of participative leadership. For example, in a research on leaders in business and government, Black (2020) showed that leaders’ self-awareness has a significant impact on their leadership style, and the higher the level of self-reported individual awareness, the more pronounced the participative leadership style. In addition, Somech’s (2003) research (2003), in conjunction with the leader-member exchange model, suggests that individual differences between leaders and subordinates also influence leadership style, the greater the differences, the less likely the leader is to implement participative management. In other words, the quality of the relationship between the leaders and the subordinates may influence the leaders’ management style. On this basis, Chen and Tjosvold (2006) also confirmed the idea that leader-member exchange quality is a key influence on participative management. The study further points out that cooperation, compared with competitiveness and independence, is an important basis for high-quality leader-member exchange, and the resulting leader-member relationships improve individual confidence and overcome cross-cultural differences, thus effectively enhancing participative management.

#### Organizational-Level Antecedents

Based on existing research, it is easy to understand the important role that personal factors play in predicting leadership styles in managerial roles. However, there can be significant differences in the way individuals lead in different contexts, as individuals in different situational organizations actively socialize by choosing to behave in a way that matches the context in which they are placed. There is no doubt that organizational context becomes a key factor in influencing leadership behaviors and styles (Schneider, 1983). For example, leaders in small-scale societies living in primitive nomadic, hunter-gatherer societies were particularly focused on participative decision-making management, whereas in the era of intensive agricultural societies, as group size increased, participative decision-making management in small-scale societies often became ineffective, while increased social complexity and distortions in the distribution of power made organizational leaders rarely demonstrate participative management and instead gave rise to directive leadership (Lonati, 2020). At the same time, an organizational culture that is acceptable and supportive of participative management in the workplace is also key to the development of participative leadership (Huang et al., 2011). Bullough and De (2015) also analysed this in-depth and state that the social environment significantly increases the effectiveness of participative leadership based on the implicit leadership theory of cultural identity.

### Mechanisms of Participative Leadership

We find that participative leadership, based on different theories from the social sciences, has significantly different effects on organizational employees through different mechanisms (mediators and moderators). First, based on social exchange theory, participative leadership influences employees by promoting their job prosperity and mutual help behavior (Usman et al., 2021). Second, conservation of resources theory suggests that participative leadership would change employee behaviors in two different ways, increasing employee workload and improving organizational self-esteem (Peng et al., 2021). Third, research based on social cognitive theory confirms that participative leadership increases employees’ self-efficacy and psychological security, which in turn affects employees’ innovation and performance (Zou et al., 2020). Fourth, social information processing theory implies that the process of participative leadership affecting employee behaviors may be influenced by cultural values and other aspects (Zhang et al., 2011). Fifth, drawing on implicit leadership theory, leaders’ information-sharing behaviors can moderate the relationship between participative leadership and employee performance (Lam et al., 2015).

#### Social Exchange Theory

Social exchange theory has become an essential theory in researching the relationship between leaders and subordinates’ work attitudes and behaviors (Miao et al., 2014). Some scholars had pointed out that Leader-Member Exchange (LMX) is to some extent reciprocal, and that supportive behaviors by the leader in an exchange relationship makes the subordinate feel obliged to reciprocate with positive attitudes and behaviors. In this way, social exchange theory, to a certain extent, provides a powerful explanation for participative leadership research. Because participative leaders encourage employees to express their personal views and opinions, actively give them the power to make decisions about their work, more respect and information resources to facilitate their participation in organizational decision-making, these signals of concern and support lead employees to perceive favors from their leaders, which in turn leads them to adopt a series of behaviors in return for their leaders (Xiang and Long, 2013). Despite the uncertainty of social exchange, most subordinates will respond positively to the participative management behaviors of their leaders based on the normative principle of reciprocity. Because the process of leaders consulting employees before making decisions makes a positive social exchange relationship, employees tend to perform better at work. Based on social exchange theory, Usman et al. (2021) also confirmed that employees encouraged by participative leadership behaviors performed better in terms of job prosperity and took the initiative to offer help to others.

#### Conservation of Resources Theory

COR recognizes that individuals have limited resources and that personal resources must be acquired, preserved and maintained on an ongoing basis. “Resources” is a broad term that includes not only the objects (e.g., pay), conditions (e.g., organizational status) and energy that individuals value in achieving their goals, but also individual characteristics. Of these, individual characteristics are seen as important resources that further influence how employees deal with other changes in their resources (Hobfoll and Shirom, 2001). For example, participative management may lead to higher performance goals for highly committed employees and less effort for less committed employees to conserve resources (Benoliel and Somech, 2014). That is, different individuals hold different amounts and types of available resources and respond differently to the problems they face in work. It is important to note that, according to resource conservation theory, individuals are naturally motivated to acquire and maintain the resources that are more important to them. And as a result of this motive, individual resources may undergo two distinct changes in resource gain or resource loss, where resource gain indicates that the initial resource gainer is more capable of acquiring the resource, and resource loss refers to an initial threat to the resource that tends to lead to increased resource loss (Halbesleben et al., 2014). Therefore, Peng et al. (2021) specifically highlighted that, according to resource conservation theory, participative leaders may have different impacts on employee resources through the two pathways described above. First, participative management provides employees with certain resources, resulting in various degrees of increase in employees’ sense of value and self-esteem, thus triggering resource gains. Second, participative management adds extra workloads to employees, thus triggering resource losses. In conclusion, resource conservation theory reasonably explain the effect of participative leadership on subordinates’ work behaviors.

#### Social Cognitive Theory

Social cognitive theory has found that the external environment, cognitive factors and individual behavior interact with each other, and individuals adjust their cognition according to the information they receive from the external environment, so as to display and maintain behavior patterns that match their own cognition (Bandura, 1978). That is, people can learn indirectly by observing, accurately perceiving the behavior of others and extracting information from it. And in leadership research, employee behavior is a product of perceptions of the environment. As a specific external environment, the messages conveyed by participative leadership style are an important part of employees’ daily contact in the workplace, and by observing and interpreting such messages, employees would change their perceptions of their own abilities and thus adopt behaviors that are consistent with them (Zou et al., 2020). For example, research by Fatima et al. (2017) based on social cognitive theory finds that participative leadership, as one of the important environmental factors, is easier for employees with higher achievement needs to access environmental information and to apply and transform it during the influence of participative leadership on the creativity of their subordinates. Furthermore, within the research framework of the environment-cognition-behavior, participative leadership has been found to be effective in enhancing employees’ self-efficacy (perceptions of self-efficacy) and psychological security (perceptions of the interpersonal environment), contributing significantly to employees’ innovation and performance (Zou et al., 2020). There is no doubt that social cognitive theory provides a new theoretical perspective and research framework for understanding the influence of participative leadership on employee behavior.

#### Social Information Processing Theory

SIP is concerned with the influence of the work environment on individual behaviors and work outcomes. It aims to reveal that individuals in organizations with a high degree of environmental adaptability actively or passively acquire information from the internal environment and process it according to certain rules to control their own attitudes and behaviors (Gao et al., 2021). And SIP effectively explains individual behavioral change and provides a solid theoretical basis for describing participative leaders’ implementation of participative management. For example, research based on social information processing theory emphases that subordinates’ perceptions, beliefs and attitudes are influenced by information about their surroundings, such as values, norms and expectations from society (Zhang et al., 2011). Leaders, in turn, are a key source of information for employees to access, and this information will collectively shape employees’ beliefs. That is, from a social information processing perspective, repeated observations of the leader’s style can enable employees to construct participative decision-making behaviors that the leader appreciates and encourages (Odoardi et al., 2019). Further, research on this theory has found that participative decision-making not only informs employees about the occurrence of behaviors, but even facilitates the transformation of attitudes toward work (Somech, 2010). It is important to note, however, that when cultural values differ, individuals may weigh information that encourages participation in decision-making and thus increase or decrease the impact of such information on their work (Zhang et al., 2011). In particular, the impact that participative management by leaders may have on employees is particularly significant in large-power-distance cultures. It is easy to see that participative management messages originating from the leader are likely to be socially constructed among group members so that employees will agree on the process of working in a particular domain environment and thus adopt organizationally supported behaviors (Odoardi et al., 2019).

#### Implicit Leadership Theory

The implicit leadership theory, derived from cognitive psychology, emphasizes the expectations and beliefs of employees about the competencies that leaders should possess, and is an “internal label” that distinguishes leaders from non-leaders, effective leaders from ineffective leaders (Lu et al., 2008). In summary, leadership effectiveness in the study of implicit leadership theory does not emphasis the outcome of leadership behavior or focus on the control of situations, but exists in the minds of subordinates as a schema of their perceptions of the leader. Furthermore, if the participative leadership does not send out strong enough signals to stimulate employees to participate in decision-making in line with expectations of participative management, this can prevent the activation of the “participation model” in subordinates. In such cases, employees are more inclined to stick with the *status quo* and do not respond positively to the participative leader until they perceive that the leader’s participative behaviors have reached a certain threshold level (Lam et al., 2015). It has also been suggested that organizational culture is likely to change the effectiveness of participative leadership, as individuals influenced by their environment shape leader’s expectations, while research based on implicit leadership theory provides insight into how individual perceptions influence effective leader’s behaviors (Bullough and De, 2015). This not only reflects the important role of the theory in participative leadership research, but also provides a sound framework for a better understanding the cross-cultural organizational behavior (Huang et al., 2011).

### The Consequences of Participative Leadership

Compared to the antecedents of participative leadership, the consequences can also be divided into the individual level and the organizational level. A lot of studies have focused on employee organizational commitment and voice behavior and so on at an individual level (Miao et al., 2014). In particular, some scholars had found that the participative leadership is positively related to employee mental health, voice behavior, and creativity (Somech, 2010; Fatima et al., 2017; Usman et al., 2021). In addition, the participative leadership improves performance and innovation at the organizational levels (Kahai et al., 2004; Yan, 2011).

#### Individual-Level Outcomes

The impact of participative leadership on subordinates stems from the leader’s empowerment and the consequent changes in psychology, attitudes, behaviors and outcomes of employees. First, on the psychological front, numerous studies have shown that participative leadership is beneficial to the psychological well-being of an organization’s employees. However, over-reliance on participative management by leaders can also have a negative impact on employees to some extent. In particular, the increased work challenges and responsibilities associated with participative management at work can be more or less burdensome for some employees, resulting in psychological stress (Benoliel and Somech, 2014). Second, in terms of attitude, because participative leadership makes subordinates feel psychologically empowered, it increases the organizational commitment of some employees and even shows complete emotional trust in the leader (Miao et al., 2014). However, it is essential to note that participative leadership has no significant role in influencing employees’ perceived trust. Then, in terms of behavior, Sagnak (2016) noted that leaders who implement participative management significantly increase employees’ change-oriented organizational citizenship behaviors by motivating their subordinates, such as helpfulness among employees at work (Usman et al., 2021). In addition, participative leadership has been a significant contributor to the organizational focus on employee innovation and voice building, and has been supported by numerous scholars (Xiang and Long, 2013). Finally, in terms of outcomes, existing research suggests that participative leadership plays an important role in both the increase in employee performance and the improvement of individual competencies. In terms of current research on job performance, there has been a great deal of scholarly attention paid to subordinate work outcomes and indirectly related job prosperity (Somech, 2010; Usman et al., 2021). And on individual employee competencies, creativity has become the focus of the work of some scholars in participative leadership research (Fatima et al., 2017).

#### Organizational-Level Outcomes

Overall, participative management is gradually becoming an important management initiative for current organizational management practitioners, and participative leadership is undoubtedly a key leadership style that cannot be ignored in leadership research. And most scholars agree that participative leadership has a catalytic effect on organizations. For example, some scholars had analyzed that participative leadership significantly improves organizational performance and innovation (Kahai et al., 2004; Yan, 2011). Further, and this is confirmed by Somech’s (2010) research (2010) based on the education sector, participative management has a clear driving effect on the organizational performance in higher education. However, the positive effects of participative leadership are inevitably accompanied by some negative effects (Peng et al., 2021). In this regard, Li et al. (2018) argued, by comparing research on directive leadership, that while participative leadership has a positive impact on organizational creativity, it reduces organizational effectiveness to a certain extent. It is easy to see that the impact of participative leadership style on the organizational level is somewhat unique and complicated. In addition, numerous studies have shown that there may be a series of mediating or interacting effects of participative leadership on organizational performance and corporate capabilities (Kahai et al., 2004; Yan, 2011). Among the various research on the effects of participative leadership, it’s particularly critical to emphasize that the fact that participative leadership affects organizations by influencing employees at the individual level has become a consensus in current theoretical research and has prompted a large number of scholars to conduct in-depth studies on the subject (Kim and Schachter, 2015).

## Future Research

At present, whether in management practice or theoretical research, there is still a large research space for participative leadership, which needs to be further explored by scholars. Therefore, we prospecte and incorporate some views into the analysis framework (shown in Figure 1).

First, most of the existing literature on this leadership style is based on some of the questions in research on empowered leadership, and is still in use today (Arnold et al., 2000). However, the measurement of participative leadership is rather general, focusing on characteristics and behaviors, and lacks a deeper exploration of the psychological dimension (Arnold et al., 2000). With the development of the information technology and the continuous changes in leadership practice, the existing research has not formed a new understanding of the content of the participative leadership style, either in terms of the form of participative leadership or its measurement, so that the development of the theory is difficult to match the current leadership management practice, and some scholars had even appeared to be critical of participative leadership (Gwele, 2008). In other words, previous interpretations of participative leadership have hindered the future research and application of this theory. It is easy to see that the conceptual content of participative leadership theory still has a lot of space to be added and optimised, and that subsequent research needs to take a more comprehensive view of the theory. Therefore, there is an urgent need for theoretical research on participative leadership to be further summarised through more scientific and rigorous analytical methods, such as experimental methods, in order to effectively classify the dimensions of participative leadership according to its modern manifestations and to develop a more mature scale for the measurement of constructs.

Second, previous research suggests that participative leadership might be seen as a rational response by leaders to organizational decisions and employee needs (Zhao et al., 2019). However, participative leaders may also be subject to both internal and external pressures to implement participative management. As research in self-determination theory has shown, individual motivation is divided into autonomous motivation and controlled motivation. Whereas autonomous motivation refers to the individual’s action as a result of matching the activity with his or her values, goals, etc., control motivation emphasizes the behavioral activities that the individual is forced to take as a result of external pressures (Gagné and Deci, 2005). Therefore, the antecedents of participative leadership can be studied in detail in the future based on self-determination theory. On the one hand, the influence of individual values, goals and interests on their own management behaviors is analyzed in the light of autonomous motivation; on the other hand, the dual pressure of the internal environment (e.g., professional managerial system) and the external environment (e.g., market uncertainty) places high demands on the scientific and accurate decision-making of leaders, which undoubtedly increases their motivation to control and thus to take part in management in order to avoid the risk of dictatorship that could lead to major risks or losses. At the same time, the theory of planned behavior suggests that individual behavior is determined by their own intentions and perceptual behavioral control (McEachan et al., 2011). Some scholars have found that individual behavioral intentions are positively influenced by their behavioral attitudes, subjective norms and perceived behavioral control, respectively. That is, they are more likely to engage in participative management if leaders maintain an optimistic attitude toward it, have the support of their employees and believe they can successfully implement it. This suggests that the theory of planned behavior also plays a key role in the antecedents of participative leadership research.

Third, throughout the current research on the results of participative leadership, many scholars have paid attention on the effects at the individual level, such as happiness at work, employee performance, etc. (Chen and Tjosvold, 2006). And there is still more room for research on the analysis of results relative to the organizational level, especially on aspects such as organizational change. As a particular form of group decision-making, participative leadership may have a beneficial effect on smaller organizational changes. However, when faced with large organizational changes, employees may be concerned about career risks and may be a deterrent to smooth organizational change in the process of participation in decision-making. Moreover, much of existing research has focused on the positive effects of participative leadership. However, the too-much-of-a-good-thing effect (TMGTE) also plays a key role in organizational leadership research and cannot be ignored. This effect suggests that over-implementation of a behavior is likely to have potentially negative influences. From this perspective, leaders who practice high levels of participative leadership and over-empower employees to participate in organizational decision-making can lead to the TMGTE. In particular, the dual-task processing effect, whereby participative leaders delegate more power or tasks to subordinates in organizational decision-making, significantly increases the amount and variety of work performed by employees, and reduces employee well-being (Peng et al., 2021). Therefore, a deeper analysis of the formation mechanism of the negative effects of participative leadership can be carried out, and a theoretical framework on the motives, concrete manifestations and path mechanisms of its behavior can be systematically constructed, with a view to providing strategies and suggestions for leaders to make scientific and practical decisions.

Fourth, both management practice and academic studies suggest that participative leaders’ management may be more likely to attract individuals with higher motivation and values to join the organization and, by effectively enhancing the identity of the organization’s members, to successfully implement participative management initiatives, which in turn may evolve into a more integrated and holistic decision-making mechanism covering all employees of the organization (Odoardi et al., 2019). Thus, future research could analyze the mediating effect of organizational identity in the relationship between participative leadership and influence effects based on social identity theory, and further explore other aspects of mediation mechanisms. It’s also worth noting that the relationship between participative leadership and subordinates’ behavioral performance is also influenced by a number of variables, in particular the organizational context (e.g., systems and culture) and individual differences (e.g., subordinates’ regulatory orientation characteristics). As most organizations are now actively building workplaces that attract and retain employees, and as organizations flatten, the culture and systems are more participative, the idea of employee participation in organizational decision-making is being accelerated at all levels of the organization (Somech, 2010; Lythreatis et al., 2019). In addition, if employees exhibit promotion focus (prevention focus), they may maintain a positive (negative) attitude toward the leader’s participative management, which also affects to a certain extent the effectiveness of the leadership participative management when implemented. In conclusion, the exploration of the intrinsic mediating mechanisms and boundary conditions of the effects of participative leadership is conducive to revealing the operational mechanisms and mechanisms of action of participative management, promoting the integration of relevant factors into a more unified framework and enriching the theoretical research of participative leadership.

Finally, as a type of democratic leadership style, although participative management has attracted the attention of some Chinese scholars. However, influenced by China’s thousands of years of history and culture, long-term authoritarian rule has caused individuals to lack a sense of independence, and employees have shown dependence and submissiveness to their leaders. Therefore, participative leadership has not received much attention from Chinese scholars. However, as the new generation of employees, such as the post-90s generation and post-00s generation, is flooding into various positions in enterprises and institutions, more and more employees are showing strong values of independence and freedom. The practice has also shown that the new generation of employees is active, receptive to information and innovative, and that participation in management not only helps to avoid the negative emotions of employees due to the dictatorship of the leader, but also facilitates the absorption of new ideas and information by the leader, and produces innovative results, which proves the urgent need for participation in leadership in the Chinese society. This is an important signal for Chinese scholars to localize the researches of participative leadership in the context of Chinese society, as western thought is constantly impacting on traditional Chinese culture and organizations in western countries are placing more emphasis on participation in decision-making than China, and are actively taking several measures to this end. Although empirical research on participative leadership has started to gradually increase in recent years, there is still more room for development (Zou et al., 2020). For example, research related to differential leadership based on the question of whether there are differences in the rights of participative leaders to involve different subordinates in organizational decision-making. In particular, leaders who have long been influenced by traditional Chinese culture are prone to self-perception based on closeness of relationships and classify subordinates as insiders and outsiders, resulting in significant differences in access to decision-making authority for different employees.

## Conclusion

As the market becomes increasingly competitive, it is difficult for leaders to make effective decisions independently. As a result, participative leadership is becoming an important element in leadership research. Scholars are also aware of the need to implement participative management in organizational decision-making. In terms of current theoretical research, there are elements of participative leadership that can be further developed and explored. From the perspective of management decisions in practice, participative leadership has dramatically improved the effectiveness of leadership decisions. This study systematically sorts out the concept and measurement of participative leadership and compare it with empowering leadership and directive leadership. We not only discuss the antecedents and outcomes of participative leadership, but also provide an in-depth analysis of the mechanisms by which participative leadership influences employees based on social exchange theory, social cognitive theory, resource conservation theory, implicit leadership theory, and social information processing theory. Finally, we propose a framework for future research on participative leadership that encompasses five potential research areas, including connotation, antecedents, outcomes, mediators and moderators, and study of participative leadership in China.

Through a systematic review of research related to participative leadership, this study makes several contributions to the development of participative leadership as follows. First, we clarify the concept, measurement, antecedents, theoretical foundations, and results of participative leadership to lay the foundation for subsequent participative leadership research. Second, we systematically compare participative leadership with directive and empowering leadership, distinguish the similarities and differences among the three, and clarify the unique research value of participative leadership. Third, by reviewing previous research on participative leadership and taking into account current leadership trends, we propose several future research perspectives, thus exploring what is currently neglected by scholars.

## Author Contributions

QW mainly made important contributions in clarifying the idea of the article, selecting the research method, literature collection, and article writing. HH made substantial contributions to literature collection, article revision, and optimization. ZL mainly played a crucial role in literature collection. All authors made outstanding contributions to this research.

## Conflict of Interest

The authors declare that the research was conducted in the absence of any commercial or financial relationships that could be construed as a potential conflict of interest.

## Publisher’s Note

All claims expressed in this article are solely those of the authors and do not necessarily represent those of their affiliated organizations, or those of the publisher, the editors and the reviewers. Any product that may be evaluated in this article, or claim that may be made by its manufacturer, is not guaranteed or endorsed by the publisher.
